# Dataset reporting *BCKDK* interference in a BCAA-catabolism restricted environment

**DOI:** 10.1016/j.dib.2016.03.038

**Published:** 2016-03-15

**Authors:** I. Bravo-Alonso, A. Oyarzabal, M. Sánchez-Aragó, M.T. Rejas, B. Merinero, A. García-Cazorla, R. Artuch, M. Ugarte, P. Rodríguez-Pombo

**Affiliations:** a Centro de Diagnóstico de Enfermedades Moleculares (CEDEM), Departamento de Biología Molecular, Centro de Biología Molecular Severo Ochoa (CSIC-UAM), U-746 Centro de Investigación Biomédica en Red de Enfermedades Raras CIBERER-ISCIII, IDIPAZ, Universidad Autónoma de Madrid, Spain; b Departamento de Biología Molecular, Centro de Biología Molecular Severo Ochoa, (CSIC-UAM), U-713 Centro de Investigación Biomédica en Red de Enfermedades Raras CIBERER-ISCIII, Instituto de Investigación Hospital 12 de Octubre, Universidad Autónoma de Madrid, Spain; c Servicio de Microscopía Electrónica, Centro de Biología Molecular Severo Ochoa, Consejo Superior de Investigaciones Científicas, Universidad Autónoma de Madrid, Madrid, Spain; d Department of Neurology, Hospital Sant Joan de Déu (HSJD), U-703 CIBER de Enfermedades Raras (CIBERER), Barcelona, Spain; e Department of Biochemistry, Hospital Sant Joan de Déu (HSJD), U-703 CIBER de Enfermedades Raras (CIBERER), Barcelona, Spain

**Keywords:** BCKDK interference, Branched-chain α-ketoacid dehydrogenase, Bioenergetics profile, Unrestrained branched-chain amino acids catabolism

## Abstract

This data article contains complementary figures to the research article “Mitochondrial response to the BCKDK-deficiency: some clues to understand the positive dietary response in this form of autism” [1]. Herein we present data relative to the effect of knocking down *BCKDK* gene on the real time oxygen consumption rate of fibroblasts obtained from a Maple Syrup Urine Disease (MSUD) patient. Interference of *BCKDK* expression on such cells showing a reduced branched-chain α-ketoacid dehydrogenase (BCKDHc) activity; let us generate a scenario to study the direct effect of BCKDK absence in an environment of high branched-chain amino acids (BCAAs) concentrations. Data relative to the effectiveness of the knockdown together with the potentiality of the *BCKDK*-knockdown to increase the deficient branched-chain α-ketoacid dehydrogenase activity detected in MSUD patients are also shown.

## **Specifications Table**

**Table:** 

Subject area	*Biology*
More specific subject area	Molecular Biology, Molecular Medicine
Type of data	Text file, graph, figure
How data was acquired	qRT-PCR (LightCycler®480), XF24 Extracellular Flux analyzer (Seahorse Bioscience, Izasa Scientific)
Data format	Analyzed
Experimental factors	MSUD-patient’ fibroblasts lentiviral transduced with shRNAs against *BCKDK* gene
Experimental features	qRT-PCR, oxygen consumption ratio in intact cells, radiometric assay
Data source location	Spain
Data accessibility	Data is within this article

## **Value of the data**

• These data provide information to establish an adequate framework to evaluate the effect of the BCAA deficiency on mitochondrial functionality in human cells.
• These data are valuable for researchers using inhibitors of BCKD kinase as therapeutic approach to treat MSUD patients.
• These data could be relevant for Neurologist treating BCKDK-deficient patients and nutritionist involved in treatments with BCAAs.

## 1 Data

**Fig. 1:**
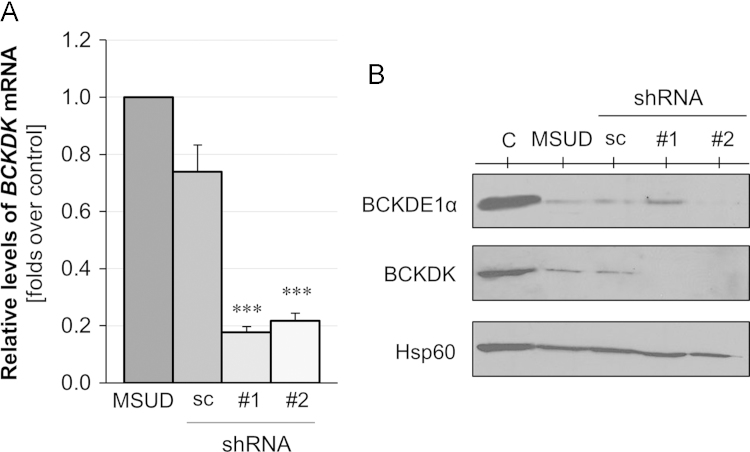
Characterization of the knockdown of *BCKDK* gene in MSUD fibroblasts. (A) Quantitative PCR analysis of *BCKDK* mRNA relative to *GAPDH* in non-transduced (MSUD) or lentiviral-transduced MSUD fibroblasts (MSUD shRNAs). Non-target control shRNA (sc); shRNA1 (#1); shRNA2 (#2) were used to prove the degree of genetic expression interference. (B) Representative Western blot of mitochondrial extracts blotted against BCKD kinase (BCKDK) to assess the BCKDK protein expression; BCKDE1α to evaluate the presence of BCKDE1α; and Hsp60 as a loading control.

**Fig. 2:**
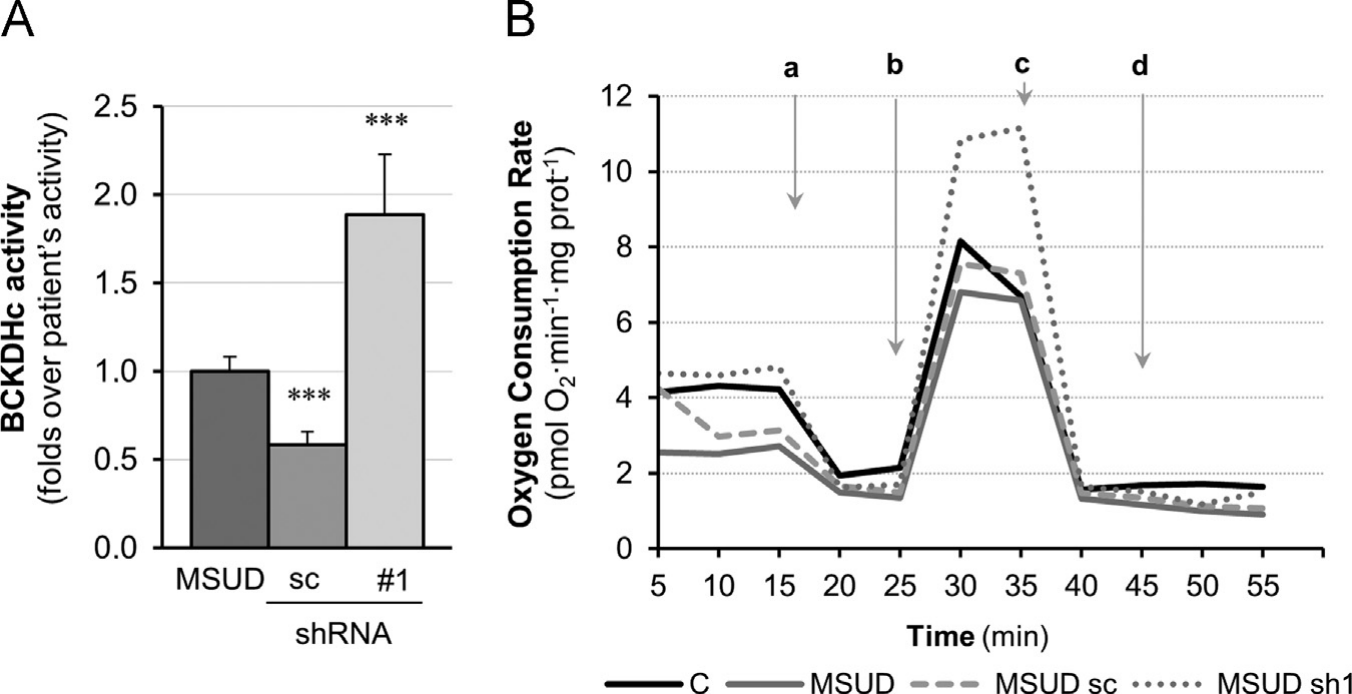
Knockdown of *BCKDK* gene in MSUD fibroblasts. Analysis of BCKDHc activity and bioenergetics profile. (A) BCKDHc activity was measured in MSUD fibroblast׳s extracts (MSUD); or after lentiviral-transduction with non-target control shRNA (sc) or shRNA1 (#1). Histogram represents the folds increases over basal. (B) Oxygen Consumption Rate -OCR- was measured as an evaluation of mitochondrial respiratory profile changes in control cells (C), non-transduced (MSUD) or lentiviral-transduced MSUD fibroblasts with non-target control (sc) or shRNA1 (sh1). Results are the mean±SD of 3–5 wells from *n*=2 independent experiments.

We present data covering the characterization of *BCKDK* gene expression interference in fibroblasts from a MSUD patient carrying missense changes in the gene encoding for the BCKDHE1α subunit of the BCKDH complex, as well as the following assessment of the bioenergetics performance of such interfered fibroblasts. The evaluation of the *BCKDK* interference was assessed by measurement of *BCKDK* mRNA by real-time PCR and protein expression through Western blot analysis. Fibroblasts’ from control and MSUD-patient interfered or not with shRNAs were included (Fig. 1A and B). The BCKDH complex activity and mitochondrial performance upon interference were assessed by establishment of the [1-^14^C]-leucine decarboxylation (see Fig. 2A) and the real-time oxygen consumption rates respectively (Fig. 2B).

## 2 Experimental design, materials and methods

### 2.1 Cell lines and culture conditions

For these studies, we used skin fibroblasts from a MSUD patient carrying known mutations in the BCKDHE1α subunit of the BCKDH complex. Control cells were the human dermal fibroblasts CC2509 obtained from Lonza (Spain). Cells were cultured with minimal essential medium (MEM) supplemented with 1% glutamine, 10% fetal bovine serum (FBS), and antibiotics in a humidified atmosphere containing 5% CO_2._ Experiments were accomplished when cells reached 80% confluence and between the 8th and 13th passage. The institutional Ethic Committee of the Universidad Autónoma de Madrid granted ethical approval for the use of the patient sample.

### 2.2 Lentiviral construct and validation

Lentiviral particles incorporating shRNAs against *BCKDK* (NM_00588.1) (Mission shRNA, Sigma Aldrich, St., MO) were generated in HEK293T packaging cells by co-transfection of pLKO.1 plasmid containing shRNA sequences, packing plasmid pCMV-dR8.74, and envelope plasmid pMD2.G (Addgene, Cambridge, MA) using Lipofectamine and Plus reagent (Life Technologies, Carlsbad, CA). Medium containing viral particles (non-target control shRNA -SHC002- or either of the *BCKDK* shRNAs targeting different sequences of human *BCKDK*) were removed 48 h after transfection. Infections of MSUD fibroblasts were performed as described in [1]. ShRNA clone information: clone TRC number: TRCN0000199200, TRCN000010196, and TRCN000010183; clone ID: NM_005881.1-828s1c1, NM_005881.x-850s1c1, and NM_005881.x-135s1c1. http://www.sigmaaldrich.com/catalog/genes/BCKDK?lang=es&region=ES#shRNA.

### 2.3 Real-time PCR quantification

Success on *BCKDK* interference was evaluated through qRT-PCR using a LightCycler®480 instrument. Total RNA was isolated using MagNaPure Compact RNA Isolation Kit and MagNaPure Compact instrument (Roche Applied Science). RNA was retro-transcribed using the High Capacity RNA to cDNA kit and GeneAmpPCRSystem9700 (Life Technologies). Real-time PCR was performed in a total of 10 μl, containing 5 ng of cDNA product, 5 μl of LyghtCycler®480 SYBR Green I Master (Roche Applied Sciences), and PCR primers at final concentration of 250 nM each. *GAPDH* mRNA expression levels were used as an endogenous control. The data were analyzed using LyghtCycler®480 software (Roche Applied Sciences) correlating the initial template concentration with the cycle threshold (Ct) to obtain the relative quantity (RQ) of RNA, defined in detail in [1].

### 2.4 Mitochondrial isolation and western blot

Mitochondria from control and MSUD-patient fibroblasts lentiviral transduced or not were isolated by magnetic separation using Mitochondrial Isolation Kit human (MACS® Technology for human, Miltenyi Biotec Inc., Germany) following manufacturer instructions. Western blot analysis of the mitochondrial fraction was performed as described in [2]. The primary polyclonal antibodies used were against BCKDK (ab111716; Abcam, Spain) at a concentration of 1:1000, and BCKDE1A (sc-67200; Santa Cruz Biotechnology) at a concentration of 1:400. Anti-Hsp60 (Stressgen, Ann Arbor, MI) was used at a concentration of 1:5000 as a loading control.

### 2.5 Measurement of BCKDH activity in MSUD patient fibroblast after infection with *BCKDK* shRNA/lentivirus

The rate of decarboxylation of [1-^14^C]-leucine was measured using the Wendel modified method [3]. Cells from one 100 mm culture dish were resuspended in 200 μl of 2 mM glucose in Krebs Ringer Phosphate buffer. The complete reaction mixture (final volume 100 μl) for the enzyme assay contained: 50 μl of cell suspension, 0.1 mM L-leucine, 10 mM glucose, 0.5 μCi [1-^14^C]-leucine and Krebs Ringer Phosphate buffer. Assay proceeded during 4 h at 37 °C in a humid chamber. The activity was expressed as pmol of CO_2_ released/h/mg of protein, corrected by the incorporation of [1-^14^C]-leucine into proteins as a metabolic control [4]. The protein concentration was measured by the Lowry method [5], using BSA as a standard.

### 2.6 Plate respirometer assay: oxygen consumption measurement by Seahorse technology

Oxygen consumption rate (OCR) was measured using the XF24 Extracellular Flux Analyzer (Seahorse Bioscience, Billerica, MA). 80,000 fibroblasts per well were seeded in XF 24-well cell culture microplates (Seahorse bioscience, Billerica, MA) in 250 μl of MEM 10% FBS, and incubated at 37 °C/5% CO_2_ for 24 h. On the first step of the assay, the growth medium from each well was replaced with 700 μl of MEM pre-warmed at 37 °C. Cells were incubated at least 1 h at 37 °C to allow media temperature and pH to reach equilibrium before the first rate measurement. After an OCR baseline measurement, 50 μl of olygomicin, carbonil 4-(trifluoromethoxy) phenylhydrazone (FCCP), rotenone and actymicin solutions were sequentially added to each well to reach working concentrations of 6 μM, 50 μM, 1 μM and 1 μM respectively [6]. Once finished the OCR measurements, data were expressed as pmol of O_2_/min/mg protein. Protein of each well quantified by Bradford׳s method (Bio-Rad) after cell lysis in 1% Triton X-100, 10% glycerol, 150 mM NaCl, 10 mM Tris–HCl pH7.5 (RIPA buffer), were used to calibrate the oxygen consumption data. Parameters related to mitochondrial function could be then evaluated. Oligomycin sensitive respiration (OSR) was determined as the difference between basal OCR and the OCR obtained after addition of oligomycin. In this point the ATP production would be stopped. Injection of FCCP, an inner membrane uncoupler, will lead to an increase in the OCR showing the maximal respiratory capacity. Finally, rotenone and antimycin A, simultaneously added (inhibitors of complex I and III respectively) will shut down the mitochondrial electron transfer chain (ETC), giving raise the non-mitochondrial respiration. Mitochondrial reserve capacity could be calculated as the difference between basal respiration and maximal respiration capacity.
